# Myeloid CD169/Siglec1: An immunoregulatory biomarker in viral disease

**DOI:** 10.3389/fmed.2022.979373

**Published:** 2022-09-23

**Authors:** Silva Herzog, Paraskevi C. Fragkou, Borros M. Arneth, Samr Mkhlof, Chrysanthi Skevaki

**Affiliations:** ^1^Institute of Laboratory Medicine and Pathobiochemistry, Molecular Diagnostics, Justus Liebig University Giessen, Giessen, Germany; ^2^The European Society of Clinical Microbiology and Infection (ESCMID) Study Group for Respiratory Viruses (ESGREV), Basel, Switzerland; ^3^First Department of Critical Care Medicine and Pulmonary Services, School of Medicine, Evangelismos Hospital, National and Kapodistrian University of Athens, Athens, Greece; ^4^Institute of Laboratory Medicine and Pathobiochemistry, Molecular Diagnostics, Philipps-University Marburg, Marburg, Germany; ^5^Universities of Giessen and Marburg Lung Center, German Center for Lung Research (DZL), Marburg, Germany

**Keywords:** CD169, Siglec1, Sialoadhesin, infection, respiratory infection, immune response, respiratory virus, SARS-CoV-2

## Abstract

CD169, also known as Siglec1 or Sialoadhesin (Sn), is a surface adhesion molecule on human myeloid cells. Being part of the Siglec family, it acts as a receptor for sialylated molecular structures, which are found among various pathogenic and non-pathogenic ligands. Recent data suggest that CD169 may represent a promising new biomarker in acute respiratory and non-respiratory viral infections, such as SARS-CoV-2, Respiratory syncytial virus (RSV) and Human immunodeficiency virus (HIV). Therein lies a great potential to sufficiently differentiate viral from bacterial infection, which has been an incessant challenge in the clinical management of infectious disease. CD169 equips myeloid cells with functions, reaching far beyond pathogen elimination. In fact, CD169 seems to crosslink innate and adaptive immunity by antigen presentation and consecutive pathogen elimination, embodying a substantial pillar of immunoregulation. Yet, our knowledge about the kinetics, mechanisms of induction, signaling pathways and its precise role in host-pathogen interaction remains largely obscure. In this review, we describe the role of CD169 as a potentially novel diagnostic biomarker for respiratory viral infection by evaluating its strengths and weaknesses and considering host factors that are involved in pathogenesis of virus infection. Finally, this brief review aims to point out shortcomings of available evidence, thus, guiding future work revolving the topic.

## Introduction

The correct diagnosis of different infectious diseases in an outpatient or emergency department setting, where rapid decision making and triage are essential for disease management, is constantly being a challenge. Established biomarkers, such as C-reactive protein (CRP), procalcitonin (PCT), erythrocyte sedimentation rate (ESR), and white blood cell counts (WBC) do not provide the needed specificity to precisely differentiate and, thus, diagnose the causative microorganism; oftentimes, these biomarkers are associated with numerous other conditions, such as comorbidities, age, medication, etc. (1), and therefore they show a wide variance of their diagnostic performance (2). Researching new reliable biomarkers in the field of infectious disease would significantly improve the concept of individualized medicine. Differentiating early and accurately viral and bacterial infections will have the great benefit of reducing mortality and morbidity as well as the spread of multi-resistant pathogens due to inappropriate antibiotic use.

CD169 (Siglec1; Sialoadhesin) is featured in several infectious and inflammatory conditions, including autoimmune disease (3, 4) and organ transplant rejection (5). Both protective and pathogenic roles have been attributed to this receptor in several viral infections, e.g., involving HIV, pathogenic murine retrovirus, Zika virus, Dengue virus, and porcine reproductive and respiratory syndrome virus (PRRSV) (6–12). Recent data show that CD169 may be a potential biomarker in respiratory viral diseases, caused by SARS-CoV-2, RSV and Influenza A virus (13–22). This review aims to focus on CD169 and its diagnostic performance in acute viral infection. Potentially involved host factors, that are driving CD169 expression, will be also elaborated.

### Molecular characteristics and potential contributions in immunoregulation

CD169 is an inducible and continuously expressed cell surface molecule on mononuclear phagocyte immune cells [macrophages (Mϕ), monocytes (Mo), dendritic cells (DC)], occurring among mammalian species (23, 24), which binds to sialylated ligands [preferentially α (2, 3)-linked sialic acid glycoconjugates] on endogenous and foreign pathogenic membrane surfaces (25, 26). Its level of expression varies among cell subpopulations, depending on the tissue and the niche. (27, 28) CD169+ macrophage subpopulations are strategically situated within the secondary lymphoid organs of lymphnodes [subcapsular sinus macrophages (SSMs) and medullary sinus macrophages (MSMs)] and spleen [marginal metallophilic macrophages (MMMs)]. There, they reside near B- and T-cell populations, serving immunosurveillance. Furthermore, they are distributed extensively among tissue resident macrophage subpopulation (e.g., lung, gut, skin, liver) (29–31) CD169 is characterized by 17-Ig-like domains (23, 32), allowing for pathogen binding, mediating pathogen uptake and cell-cell adhesion between different immune cells. The cytoplasmic residue of CD169, however, is rather short and poorly conserved in length and sequence, which leads to the conclusion, that the predominant function of the receptor is primarily cellular interaction, rather than signaling (23, 24, 33) (Supplementary Figure 1).

The roles and functions in immunoregulatory processes seem to be manifold, including pathogen recognition (28), pathogen uptake (28, 34, 35) cross-presentation to CD8α^+^ DCs (T cell priming) (25, 33, 36, 37), as well as B-cell and invariant natural killer T-cell (iNKT) activation (33, 38).

It has been repeatedly demonstrated, that CD169 plays a crucial role in cases of *trans-*infection in retrovirus invasion (7, 9, 39–43). Thereby, viruses exposing sialylated gangliosides in their outer surface composition are recognized and uptaken by CD169-expressing myeloid cells and subsequently presented to susceptible CD4+ T-cells at the cell-cell interface between antigen presenting cell (APC) and T-cell (39). The ability to *trans* infect has recently been described in the context of SARS-CoV-2 infection as well, extending the results shown in retroviruses (44, 45). In a similar way to what has been described in Ebola-virus infection (46), Perez-Zsolt et al. reported the storage of SARS-CoV-2 particles within virus containing compartments (VCCs) on infected DCs, eventually resulting in *trans-*infection of ACE2-and TMPRSS2-expressing cells *via* DCs (44, 47). Treatment with monoclonal antibodies against the CD169 receptor demonstrated significant decrease of *trans-*infection (40), which shows potential as a therapeutic target.

**FIGURE 1:**
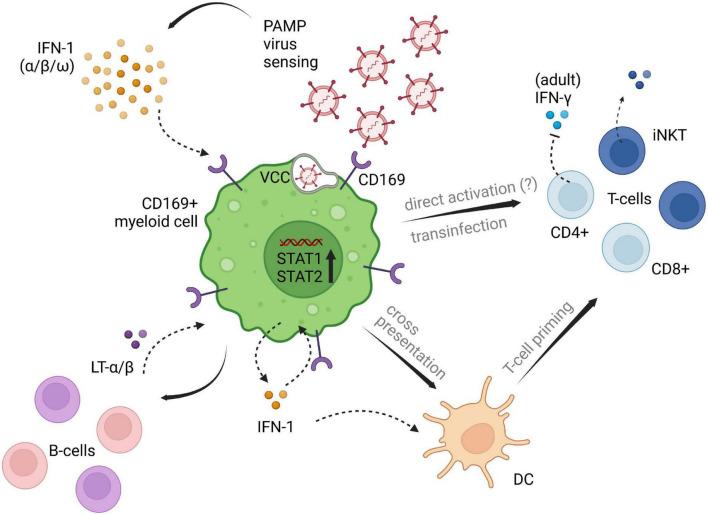
Potential implications of CD169+ myeloid cells on different cell subsets of innate and adaptive immunity. CD169+ expression is induced by type I IFN signaling following viral penetration. Downstream IFN pathways are set in motion, promoting virus titer control and adaptive immune cell priming and recruitment. Influencing factors of type I IFN induced CD169, that are contributing to the impairment of antiviral immunity need further clarification. The displayed pathways likely differ between myeloid cell lines, as well as between adults and children, and are depicted in one single figure, solely for illustrating purposes of the processes described in available literature. PAMP, pathogen associated molecular patterns; LT-α/β, lymphotoxin α/β; IFN, interferon; DC, dendritic cell; VCC, virus containing compartment. The figure was created with BioRender.com.

Pediatric studies are rather scarce. However, Jans et al. investigated the *in vitro* monocytic CD169 expression levels in infants and adults following RSV infection and found comparable upregulation among patient groups. Their research demonstrated, that CD169 induced through RSV infection led to a decrease of interferon-γ (IFN-γ) release by adult CD4+ T-cells, whereas the infant group showed no reduction of IFN-γ, possibly due to the lack of differentiated CD4+ memory T-cells in newborn and infants. Also, lower CD43 expression on naïve T-cells were seen in the pediatric group in comparison to adult T-cell expression (22). CD43 has been proposed as the main counter receptor for CD169 on T-cells and is highly represented on adult memory T-cells (22, 48). Therefore, low CD43 on infants’ naïve T-cells is presumably accountable for the lack of inhibitory IFN-γ signaling by CD169 induction (22). An overview of potential immunoregulatory implications of CD169 is depicted in Figure 1.

Notably, CD169 is also involved in infection with certain bacteria in diverse peripheral tissues, such as skin, brain, and gut tissue. Encapsulated bacteria like *Escheria coli*, *Campylobacter jejuni*, *Neisseria meningitidis* and group A and B *Streptococcus* (GAS, GBS) have evolutionary evolved remarkable escape strategies by mimicking host polysaccharide structures like GM1. These strategies dampen anti-bacterial activities, e.g., by binding to inhibitory members of the Siglec1-family (49, 50). CD169, in contrast, is lacking inhibitory signaling motifs. CD169 is rather capable of recognizing sialylated pathogens, such as GBS, mediating phagocytosis and promoting bactericidal activities (50). In the autoimmune disease Guillain-Barré – Syndrome, *Campylobacter jejuni* expresses lipooligosaccharides structures on its surface, that are structurally similar to gangliosides in humans, allegedly causing auto reactive antibodies *via* CD169-Sia interactions (26). On the other hand, very recent evidence showed, that treatment with *Staphylococcus aureus* on CD169-deficient mice indicated impaired local immune response and adaptive immune cell recruitment of IL-17 producing γδ T-cells in the dermis (51). Additionally, an increase in bacterial burden was observed and the authors proposed a local type I IFN signaling pathway driving CD169 expression, but the source of type I IFN could not be elucidated (51). Lastly, results from knockout experiments on CD169 in *Mycobacterium tuberculosis* infection suggest that the receptor is responsible for the retainment of extracellular vesicles deriving from infected cells resulting in early antiviral immune activation (52).

Summing up, CD169, expressed on myeloid cells comprises fundamental implications on both innate and adaptive immunity. The profound role in pathogen recognition *via* sialylated gangliosides and consecutive virus containment has become evident (27). Beyond that, direct and indirect immunoregulatory influences on adaptive immune cell subsets in an activating, priming, and recruiting fashion are observed. Along with protective antiviral attributions, viral hijacking of CD169+ DCs with subsequent *trans-*infection of susceptible adaptive immune cells have extensively been investigated in retrovirus infection and lately been shown in SARS-CoV-2 infection. The role in enveloped virus transmission *via* CD169 mediated *trans-*infection needs future investigation and holds space for potential therapeutic targeting. Prompt CD169 upregulation on infant monocytes due to RSV infection suggests an inborne mechanism following evolutionary conserved pathogen recognition receptor (PRR) signaling.

### Mechanisms of induction and cell population maintenance

One of the main drivers of CD169 upregulation is through mechanisms that lead to type I interferon (IFN-I) responses. Especially IFN-α plays a major part in CD169 expression (23). The typical involvement of type I interferons are conditions of proinflammatory, immunoregulatory and anti-tumoral nature, which explains the increased expression of myeloid CD169 in this context (53). Furthermore, the capability of detecting and engulfing viruses is critically dependent on the recognition of sialic acid, which has been demonstrated in *in vitro* SARS-CoV-2 experiments, where mutation of CD169 led to impaired viral uptake. Monosialotetrahexosylganglioside (GM1) could be identified as one of the gangliosides interacting with CD169 *in vitro* (44). Although not shown in respiratory viral infection, several studies, found the sialic acid containing monosialodihexosylganglioside (GM3) to be another physiological ligand of CD169 (54–57) *In vivo* analyses revealed a direct and clear upregulation of the receptor upon IFN-I (α/β/ω) stimulation within the physiological ranges of viral infection (58). In addition, significant changes in pSTAT1 and pSTAT2, pathways that are crucially involved in antiviral activity, have been detected (58, 59). Of note, CD169+ macrophages have been found to be one of the main IFN-1 producers upon virus infection, themselves, making a self-enforcing process of IFN-mediated antiviral activity conceivable (27). Supporting that, *in vitro* IFN-α exposure of monocyte-derived macrophages (MDMs) and monocyte-derived dendritic cells (MDDCs) seems to enhance the capacity to uptake SARS-CoV-2 virus particles in contrast to non-stimulated APCs (44).

In mediating immunoregulatory functions, CD169+ cell subpopulations are susceptible toward environmental changes. To date, the evidence on milieu and cell maintenance relationship is obscure. Knockout analyses were able to identify some of the factors, that are creating an environmental niche, which is affecting the differentiation and maintenance of lymphoid tissue CD169+ macrophages: Receptor activator of nuclear factor kappa-B ligand (RANKL), member of the TNF superfamily is a key factor in controlling lymphnode organogenesis and is primarily expressed by marginal reticular cells, a stromal cell subset. RANKL deficiency in *Cd169-cre Rank*^fl/fl^** mice has resulted in quantitative decrease and impaired differentiation in subcapsular sinus macrophages (SSMs) and medullary sinus macrophages (MSMs) (60) Expanding on these results, Camara et al. further revealed in a different work, that deficiency in B-cell derived lymphotoxin α/β (LT α/β) and LTβ receptor (LTβR) also led to decrease in numbers of SSMs and MMMs and to exchange of lymphoid CD169+ macrophages (19, 60, 61) Additionally, Shinde et al. studied the correlation between tumor necrosis factor (TNF), IFN-I expression and CD169 expression in vesicular stomatitis virus (VSV) infection and they demonstrated that, the ablation of TNF receptor (TNF-R) and mucosa-associated lymphoid tissue lymphoma translocation protein 1 (MALT1) led to severe immunopathology (62). They concluded that TNF is contributing to the maintenance of CD169+ cells, resulting in early virus replication, thus promoting early antiviral immune activation. Of note, it’s suggested, that the retainment of viral antigen by CD169+ myeloid cells is enabling early antigen presentation, which could be beneficial in amplifying the adaptive immune response in early infection (62).

### Host factors in pathogenesis

Given the assumption that CD169 is representing an important immunoregulatory player in the early stages of infection and disease progression, implications of loss-of-function phenotypic variants have demonstrated the course of infection in null individuals; Martinez-Picado et al. investigated a large cohort of HIV-infected individuals comprising of two homozygous and 97 heterozygous individuals expressing a specific stop-codon variant (Glu88Ter) in the CD169 gene (40). This gene is variously found among individuals of different ethnicities (39, 40). Interestingly, no significant differences in terms of virus acquisition, viral plasma load nor disease progression could be seen, despite phenotypical differentially expressed haploinsufficiency or protein absence (40). Yet, the heterozygous subjects revealed lower virus capture abilities than the control group and the results indicated that homozygous loss-of-function variant cannot be compensated. Notably, the identification of two infected null individuals indicate, that functional CD169 is dispensable in terms of HIV acquisition (40). Similarly, another work on PRRSV infection in CD169 knockout pigs showed no significant differences in severity of interstitial pneumonia development or histopathology in CD169 deficient compared to heterozygous and wild type animals, despite the prove of productive PRRSV infection in the piglets (11). However, the ablation of *in vivo* CD169+ alveolar macrophage (AM) population in a transgenic CD-169-DTR mouse strain boosted inflammation and respiratory dysfunction in response to PR8 influenza virus infection, leading the authors to conclude, that CD169+ AMs are thoroughly involved in disease containment, especially early virus titer control, and development of disease severity (27).

Based on the above findings from different animal models and human data, genetic loss of function alterations of CD169 in viral infection provides conflicting results. Impaired virus control and disease aggravation possibly depends on the virus strain, the species being infected, individual immune status and cell line susceptibility. Future *ex vivo* and *in vivo* research need to shed light on the precise impact of receptor loss-of-function variants.

Regarding the CD169 expression upon IFN-α/β stimulation, impaired or deficient IFN-1 signaling, (as shown in critically ill COVID-19-patients), may also negatively influence the expression of CD169, and, therefore, the viral control in early stages of infection (16, 20, 63). The hypothesis of neutralizing IgG autoantibodies against type I IFN has been tested in SARS-CoV-2 infection and was proven to be a potential underlying factor in the course of critical disease. *In vivo* and *in vitro* studies, published by Bastard et al. demonstrate the presence of neutralizing type I IFN autoantibodies in about 10% of critically ill SARS-CoV-2 patients, that cause decrease in pSTAT1 in the majority of (almost exclusively male) patients (64). Thus, it would be interesting to see, if similar results are reproducible among other viral infections, how they correspond to CD169 expression levels and under which conditions external acquisition of interferonopathy and impairment of downstream CD169 expression is possible. Nevertheless, a connection seems very plausible, since these findings are in accordance with low CD169 levels in severe SARS-CoV-2 infection, as described by Doehn et al. (20). Moreover, Zhang et al. identified inborn type I IFN-associated deficiencies resulting in severe illness in the context of SARS-CoV-2 (64). Moreover, genetic interferonopathies of type I IFN, have been associated with high levels of monocytic CD169 in childhood autoimmune Aicardi Goutière syndrome (AGS) and Singleton-Merten syndrome (SMS) and therefore monocytic CD169 has been proposed as an effective screening marker (65).

## CD169: Clinical marker of viral disease

Although, numerous aspects of the positive and negative control mechanisms of CD169 expression in health and disease still need further clarification, its momentous participation in fundamental immunologic processes (such as surveillance, recognition of self and foreign, antigen uptake, containment, and presentation) has been demonstrated. Recently, multiple studies have been testing the capacities of CD169 as a biomarker in viral disease. Clinical trials, performed to date, mainly revolve around SARS-CoV-2 infection (13–16, 19–21, 58). Very few studies have indicated the role in infection by other respiratory viruses, such as RSV, Influenza A virus or Rhinoviruses (15, 22).

The ongoing clinical challenge is how to correctly identify the underlying cause when infection is suspected. The development of reliable host-based biomarkers is necessary to bridge the diagnostic gap in ambiguous clinical cases of infection. High sensitivity (negative test results; “rule-out-approach”) values are needed to not miss any patients with bacterial infection in need for antibiotics, whereas a “rule-in-approach” (positive test results), thus high specificity, is necessary in cases of viral infections (14, 66).

### CD169 as an early diagnostic marker in SARS-CoV-2 infection

Since the onset of SARS-CoV-2 pandemic in 2019, a series of both retrospective and prospective clinical studies, investigating the diagnostic performance and discriminative ability of (monocytic) CD169 as a host-based biomarker of acute viral disease have been performed.

Monocytic CD169 (mCD169) expression levels reliably detected viral infection and results [measured in mean fluorescence intensity values (MFI) or monocyte/lymphocyte ratio MFI (rMFI)] displayed high sensitivity (≥ 80%) and specificity (≥ 91%) throughout (Table 1). Worth mentioning, where data were available, mCD196 expression exceeded the sensitivity and specificity of CRP (14, 16). Moreover, CD169 and viral load (cycle threshold values and time to positive PCR) were directly associated (15, 16, 21). The comparison of mCD169 levels between patients with active SARS-CoV-2 infection and virally suppressed HIV patients under antiretroviral therapy, showed significant differences, substantiating the role of CD169 as a marker of acute viral infection (21). Bedin et al. researched the monocytic CD169 expression at the beginnings of the COVID-19 pandemic (March/April 2020) and several valuable observations were made (16): No association between the onset of symptoms and mCD169 levels on hospital admission could be detected. Interestingly, mCD169 levels were not connected to disease severity or need for ICU treatment. Other observations included positive correlations between mCD169 and IFN-α levels, which both decreased over time of hospitalization. Lastly, the retrospective analysis of anti-SARS-CoV-2-IgG responses, were present in 7 SARS-CoV-2 positive subjects and were accompanied by lower mCD169 expression, suggesting that higher levels are seen before seroconversion (16). These results are in accordance with published evidence by Minutolo et al. who showed positive correlation between the mCD169/lymphocyte ratio (RMFI) and the percentage of marginal naïve B-cells (19). Recently, a study demonstrated the longitudinal CD169 expression in COVID-19 infection, and the results demonstrated higher levels of expression in early stages and mild cases of SARS-CoV-2 infection, in contrast to significantly decreased expression in critically ill patients (20). In mild cases, CD169 expression peaked within the first 3 days post symptom onset and showed slow decrease to normal ranges within 3–4 weeks. In addition, as mentioned before, a strong correlation between viral load and CD169 expression in mild cases was seen, which interestingly, was not the case for severe disease (20). Additionally, the assessment of the monocyte landscape by t-distributed stochastic neighbor embedding analysis (t-SNE) showed, that the monocyte subpopulation in mild cases almost exclusively consisted of CD169+ clusters, which were significantly increased in mild disease, decreased in severe disease, and absent in healthy individuals (67). These findings once again are indicative of a protective role of CD169 in viral disease.

**TABLE 1:** Clinical trials indicating the diagnostic performance of monocytic CD169.

Study type	Respiratory virus strain	Variable	Sensitivity %	Specificity %	AUC	Threshold	References
Observational prospective	Influenza A RSV Rhinovirus	rMFI	95	100	0.98	5.34	(15)
Observational retrospective	Parainfluenza	MFI	85.71	100	0.97	1.58	(14)
n.a.	SARS-CoV-2	rMFI	91.7	89.8	0.92	3.3	(21)
n.a.	SARS-CoV-2	rMFI	97	80	0.95	3.51	(16)
n.a.	SARS-CoV-2	rMFI	97	92	0.925	3.01	(19)

The above listed virus strains are referring to respiratory virus strains identified in the trials and are not comprehensive of the overall identified pathogens. rMFI, lymphocyte/monocyte mean fluorescence intensity ratio; AUC, area under the curve; Ref, References; n.a., not available.

In contrast, increased CD169 expression in moderately and severely diseased patients have also been described in untreated patients presenting with severe interstitial pneumonia and SARS-CoV-2 associated multisystem inflammatory syndrome in children (MIS-C), suggesting that CD169 may be a prognostic marker for oxygen need and adverse outcomes (19, 36). Moreover, a significant over-expression of monocytic CD169 has been seen in patients admitted to the ICU due to severe SARS-CoV-2 infection (18).

Comparative immune profiling of patients presenting with or without acute respiratory distress syndrome (ARDS) in SARS-CoV-2 patients revealed a peculiar CD169 associated immune signature, which was not seen in ARDS patients negative for SARS-CoV-2. But no immune signature changes could be seen within the SARS-CoV-2-infected patients (ARDS vs. no ARDS) (68).

In summary, the clinical trials that have been conducted so far, show promising results for the diagnostic potential of CD169 as a screening tool for viral disease. However, not only studies are still scarce but also the “normal” values and limits of CD169 remain non-standardized. Regarding its prognostic and monitoring properties, the evidence is inconsistent. Prospective data should address the host factors, involved in the balancing act of CD169 expression between immune competence and immune deterioration, that are underlying asymptomatic/mild and severe disease progression.

Lastly, another constraint should be addressed: As already indicated, CD169 expression has been shown to be associated not only with respiratory viral disease, but also other (pathogenic) conditions of inflammatory or, infectious nature, e.g., autoimmunity, cancer, organ transplant rejection, bacterial infection, and various non-respiratory viral infections (3–5, 26, 40, 46, 50, 51, 53, 69). It may be assumed, that the diagnostic value of CD169 in patients affected with one or several of the above-mentioned pathologies is of limited significance.

## Discussion

The existing evidence clearly indicates the valuable role of CD169 in diverse immunoregulatory functions, particularly in effecting early infection and viral control as well as its impact on adaptive immunity. Being a downstream molecule of type I IFN signaling, various interferonopathies are likely to impair the expression of CD169, as has already been depicted in the literature. Besides, a loss of function variant affecting the CD169 molecule itself has also been described.

The good diagnostic performance of CD169 as a biomarker of acute viral disease so far, seems to be promising in both viral epidemics where high sensitivity is needed (“rule-out-approach”), and in non-epidemic scenarios where high specificity is required (“rule-in approach”). Nonetheless, clinical trials are still limited; moreover, establishing universal laboratory standards and methodological groundwork is crucial for the improvement of comparability of laboratory testing and results among future studies (52). This encompasses pre- and post-analytical issues, as well as prospectively randomized design of clinical studies and patient recruitment strategies. Most of the clinical trials to date, have been conducted among SARS-CoV-2 patients, and only a very small number of studies showed the significance of CD169 as a virus-induced surface marker in other respiratory viral infections, like influenza A virus and RSV. However, large studies focusing on different viral strains are needed to confirm these preliminary data. Furthermore, future study protocols should take under consideration demographic data including age, gender, ethnical background and comorbidities. Current data regarding CD169 expression in acutely infected patients, almost exclusively focuses on adult patients. Indeed, studying the expression in vulnerable patient groups, e.g., children, elderly, immunocompromised, pregnant, etc., would be particularly interesting, as these groups are commonly affected by severe viral infections, and therefore are more frequently in need for medical care or intervention. Besides viral infections, increased CD169 is seen in autoimmune conditions and anti-tumor immune responses. Hence, the diagnostic performance of CD169 as a biomarker of acute viral disease in these conditions needs further evaluation. What is more, effects of antiviral and anti-inflammatory properties on CD169 expression have not been investigated in depth yet. Finally, more research addressing the positive, but mainly the negative control mechanisms are needed to give a better understanding of origin and fate of these remarkable CD169 expressing myeloid cell subsets.

Finally, it should be mentioned, that this Mini Review has its limitations. Since the literature search has been focused on the implications of CD169 in respiratory viral disease, the connection between CD169 and HIV, respectively autoimmune (e.g., SLE) and cancerous disease have largely remained unconsidered. Moreover, especially with reference to SARS-CoV-2, new evidence emerges constantly. This literature search has been conducted with utmost care; within the common limitations of unsystematic narrative reviews, it aims to serve as a roundup on the available evidence on the role of CD169 within the scope of respiratory viral disease, while also directing future work in a field, that is very much in motion.

## Authors contribution

SH wrote the manuscript. SH, BA, and SM contributed to the literature search. BA revised the manuscript. CS and PF critically revised the manuscript and contributed to conceptualization and supervision of the work. All authors contributed to the article and approved the submitted version.

## Supplementary material

Supplementary Figure 1 **(a)** Domain organization of CD169: CD169 belongs to the Ig-like Siglec superfamily, hence the designation Sialic acid-binding immunoglobulin-type lectins. Siglecs are a group of cell surface molecules, which can roughly be subdivided into highly conserved Siglecs, including CD169, and CD33-related Siglecs (24, 70, 71). CD169, being one of the largests representatives of its family, consists of 17 immunoglobulin domains, that are characteristically protruding in the peripheral extracellular space. This feature distinguishes CD169 from other members of the Siglec family. Consequently, *trans-*binding and cell-cell interaction are the preliminary function of the molecule rather than *cis-*interaction [O’Neill (72), Munday et al. (73)]. Additionally, CD169 has a short cytoplasmic tail. Based on current knowledge it lacks inhibitory or activating signaling motifs. **(b)** N-terminal domain of CD169: The sialic-acid binding domain is situated within a shallow pouch of the V-set domain on the outer extracellular part of the molecule. PDB ID: 1QFP; PDB DOI: http://doi.org/10.2210/pdb1QFP/pdb [Deposition authors: May et al. (74)]. **(c)** Simplified presentation of the representative CD169 ligand GM1. GM1 has been identified as one ligand of CD169 in SARS-CoV-2, binding *via* its single N-acetylneuraminic acid (NANA). Glc, D-Glucose; Gal, D-Galactose; GalNac, D-acetyl-D-galactosamine (75). The figure was created with BioRender.com.
