# Supplementary figures and images for: Myeloid CD169/Siglec1: An immunoregulatory biomarker in viral disease
Source: Front Med (Lausanne). 2022 Sep 23;9:979373. doi: 10.3389/fmed.2022.979373 (PMC9540380; doi:10.3389/fmed.2022.979373)

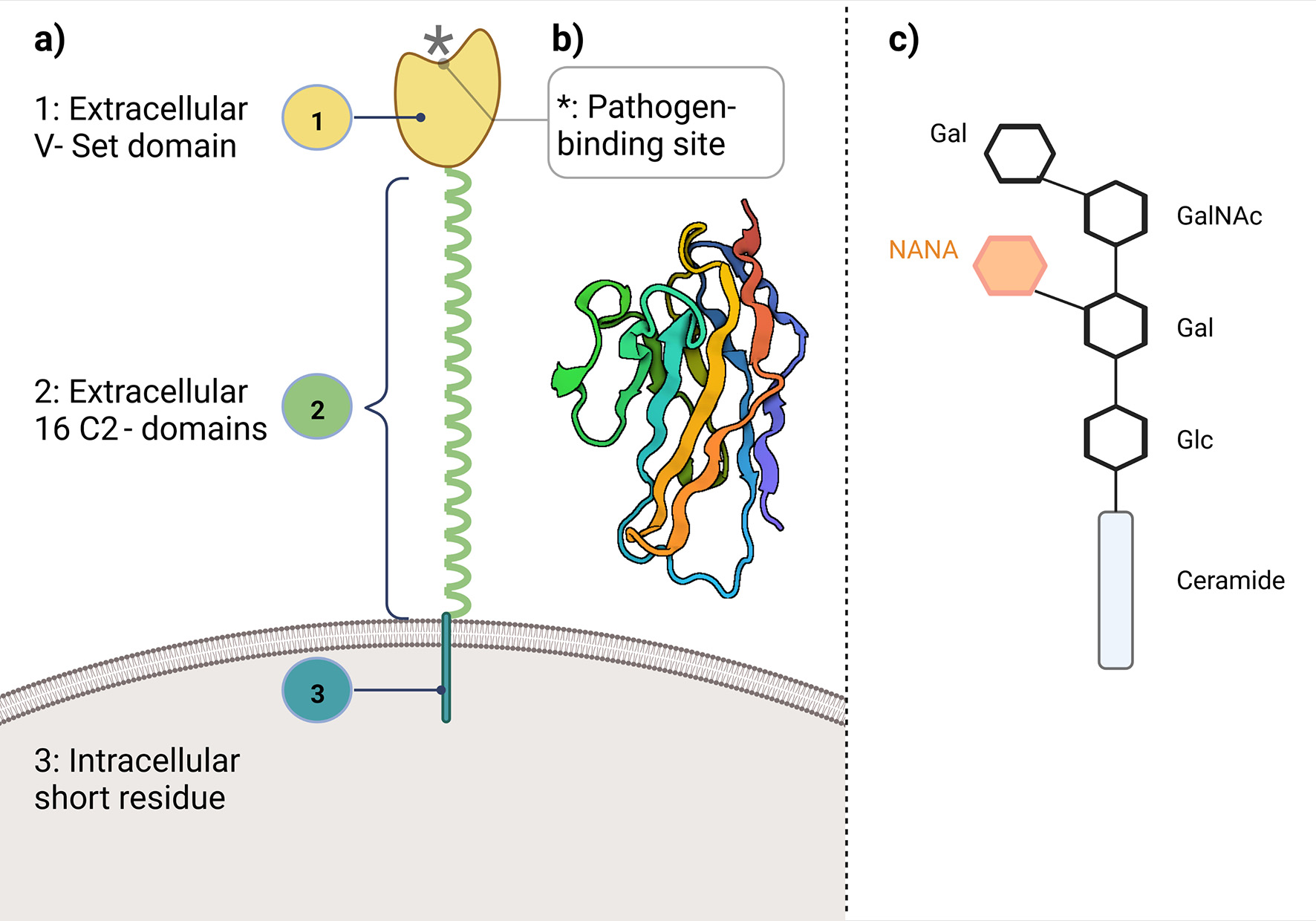

Supplement: Supplementary Figure 1 — (a) Domain organization of CD169: CD169 belongs to the Ig-like Siglec superfamily, hence the designation Sialic acid-binding immunoglobulin-type lectins. Siglecs are a group of cell surface molecules, which can roughly be subdivided into highly conserved Siglecs, including CD169, and CD33-related Siglecs (24, 70, 71). CD169, being one of the largests representatives of its family, consists of 17 immunoglobulin domains, that are characteristically protruding in the peripheral extracellular space. This feature distinguishes CD169 from other members of the Siglec family. Consequently, trans-binding and cell-cell interaction are the preliminary function of the molecule rather than cis-interaction [O’Neill (72), Munday et al. (73)]. Additionally, CD169 has a short cytoplasmic tail. Based on current knowledge it lacks inhibitory or activating signaling motifs. (b) N-terminal domain of CD169: The sialic-acid binding domain is situated within a shallow pouch of the V-set domain on the outer extracellular part of the molecule. PDB ID: 1QFP; PDB DOI: http://doi.org/10.2210/pdb1QFP/pdb [Deposition authors: May et al. (74)]. (c) Simplified presentation of the representative CD169 ligand GM1. GM1 has been identified as one ligand of CD169 in SARS-CoV-2, binding via its single N-acetylneuraminic acid (NANA). Glc, D-Glucose; Gal, D-Galactose; GalNac, D-acetyl-D-galactosamine (75). The figure was created with BioRender.com. [file Image_1.JPEG]
